# A Diagnostic Dilemma of Arrhythmogenic Cardiomyopathy Masquerading as Recurrent Myocarditis in a Pediatric Patient with a *DES* Gene Variant: A Case Report

**DOI:** 10.3390/jcdd13040162

**Published:** 2026-04-08

**Authors:** Qi Meng, Wei Li, Wenhong Ding, Hui Wang, Dong Chen, Ling Han, Yifei Li, Chencheng Dai

**Affiliations:** 1 Department of Pediatric Cardiology, Beijing Anzhen Hospital, Capital Medical University, Beijing 100029, China; qmeng2605@163.com (Q.M.); liwei0215mail@163.com (W.L.); whding9266@outlook.com (W.D.); hanl6610@sina.com (L.H.); 2 Department of Pathology, Beijing Anzhen Hospital, Capital Medical University, Beijing 100029, China; hugeren@126.com; 3 Department of Radiology, Beijing Anzhen Hospital, Capital Medical University, Beijing 100029, China; azchendong@163.com; 4 Key Laboratory of Birth Defects and Related Diseases of Women and Children of Ministry of Education, Department of Pediatrics, West China Second University Hospital, Sichuan University, Chengdu 610041, China

**Keywords:** DES, cardiomyopathy, myocardial fibrosis, myocarditis, hot-phase cardiomyopathy

## Abstract

Background: Arrhythmogenic cardiomyopathy (ACM) is an inherited disorder characterized by fibrofatty replacement of cardiomyocytes. The inflammatory episodes of ACM, known as the “hot phase”, can mimic acute myocarditis. It was seldom observed in a *DES*-associated ACM as a “hot-phase” presentation. Case Presentation: The proband, a 13-year-old female, initially presented with a series of clinical manifestations of fulminant myocarditis. Although recommendation-guided anti-immunotherapy had been provided, this patient still developed into an aggressive cardiomyopathy with biventricular dilation and severe systolic heart failure. Additionally, cardiac magnetic resonance demonstrated circumferential late gadolinium enhancement in left ventricular myocardium with diffuse fibrosis. Whole-exon sequencing identified a de novo missense variant, as c.335T>A (p.L112Q) of the *DES* gene, resulting in protein dysfunction. And a diagnosis of ACM due to a *DES* variant had been identified. Finally, this patient received heart transplantation, and biventricular fibrofatty infiltration was confirmed by pathological analysis. Conclusions: This case presented a de novo genetic variant that can induce severe and aggressive heart failure. This finding emphasizes the importance of comprehensive genetic analysis in patients suspected of having fulminant myocarditis, which would greatly benefit the precise clinical management and outcomes.

## 1. Introduction

The diagnosis of arrhythmogenic cardiomyopathy (ACM) remains clinically challenging, with misdiagnosis and diagnostic delays occurring frequently, particularly in cases with predominant left ventricular involvement [1,2,3]. These diagnostic delays are associated with increased mortality during long-term follow-up. Myocarditis represents one of the most common initial misdiagnoses due to phenotypic overlap during acute inflammatory episodes, termed “hot phases,” which are characterized by myocarditis-like clinical presentations [4,5,6,7]. Despite the substantial literature regarding the potential role of inflammation in ACM pathogenesis and progression, comprehensive clinical studies examining large cohorts of patients experiencing “hot phases” remain limited. Consequently, the true incidence of hot-phase phenomena among ACM patients remains undefined [6,8,9,10,11].

The precise molecular pathways underlying inflammation-mediated ACM progression remain incompletely characterized [12]. Critical questions persist regarding whether myocardial inflammation represents a primary pathogenic insult leading to cardiomyocyte injury or constitutes a secondary response to programmed cell death. Further mechanistic investigation is warranted to elucidate these complex pathophysiological relationships. Herein, we presented a rare case that carried a de novo variant of *DES* demonstrating complicated clinical manifestations, such as fulminant myocarditis, and, finally, diagnosed as a “hot-phase” ACM, providing the consideration of molecular assessment in distinguishing complicated cardiovascular diseases.

## 2. Methods

### 2.1. Ethical Considerations

This study was approved by the ethics committee of Beijing Anzhen Hospital at Capital Medical University. Written informed consent was obtained from the patients’ parents for whole exome sequencing (WES) and inclusion of clinical and imaging details in the publication.

### 2.2. Genetic Testing

Peripheral blood samples were collected from the proband and her parents in ethylenediaminetetraacetic acid (EDTA) anticoagulant tubes and stored at 4 °C for less than 6 h. DNA extraction was performed using the Blood Genome Column Medium Extraction Kit (Tiangen Biotech, Beijing, China) according to the manufacturer’s instructions.

WES was conducted using the NovaSeq 6000 platform (Illumina, San Diego, CA, USA). Raw data were processed using FastP v 1.0.1to remove adapters and filter low-quality reads. Paired-end reads were aligned to the Ensembl GRCh38/hg38 reference genome using the Burrows–Wheeler Aligner. Variant annotation was performed in accordance with database-sourced minor allele frequencies (MAFs) and practical guidelines on pathogenicity issued by the American College of Medical Genetics. MAF annotation was based on the 1000 Genomes, dbSNP, ESP, ExAC, and Chigene in-house MAF databases. Provean, Sift, Polypen2_hdiv, and Polypen2_hvar databases were used for further annotation using R software v 4.2.1 (R Foundation for Statistical Computing, Vienna, Austria).

### 2.3. Protein Structure Analysis

The SWSIS model tool (https://swissmodel.expasy.org/, accessed on 13 December 2025) was employed to establish protein crystal structures and analyze the mutant site of DESMIN. Pymol software v 2.5.4 was used to illustrate the molecular structures of wild-type and mutant forms of the targeted genes.

## 3. Clinical Description and Molecular Result

### 3.1. History of Illness and Physical Examination

A 13-year-old female presented to our department with recurrent syncope during prolonged standing and showering within the previous 6 months. Also, this patient complained of progressive activity intolerance and fatigue in the most recent 2 months. Moreover, the patients reported repeated experiences of chest pain several times a day. Along with such manifestations, SARS-CoV-2 infection had been identified, while the local clinic provided healthcare management according to the potential diagnosis of post-COVID-19 syndrome. In addition, the patient’s medical history did not document any positive records. Physical examination revealed a potential dysfunction of the heart. Respiratory assessment demonstrated decreased left lung breath sounds with prominent coarse crackles, predominantly in the left field. Cardiac examination revealed irregular rhythm with diminished heart sounds and significant murmurs at the mitral valve areas. The abdomen was soft with mild distention and hepatomegaly extending 2 cm below the costal margin; the spleen was non-palpable. Peripheral examination showed mild pitting edema or digital clubbing, with preserved peripheral perfusion and normal digital temperature. The family history had been carefully evaluated. Although her grandma underwent a sudden cardiac death at 79 years of age. In addition, other family members, including her parents and sister, were absent from any cardiovascular diseases, with negative results from ECG and echocardiography screening.

### 3.2. Laboratory and Imaging Evaluation

**Figure 1:**
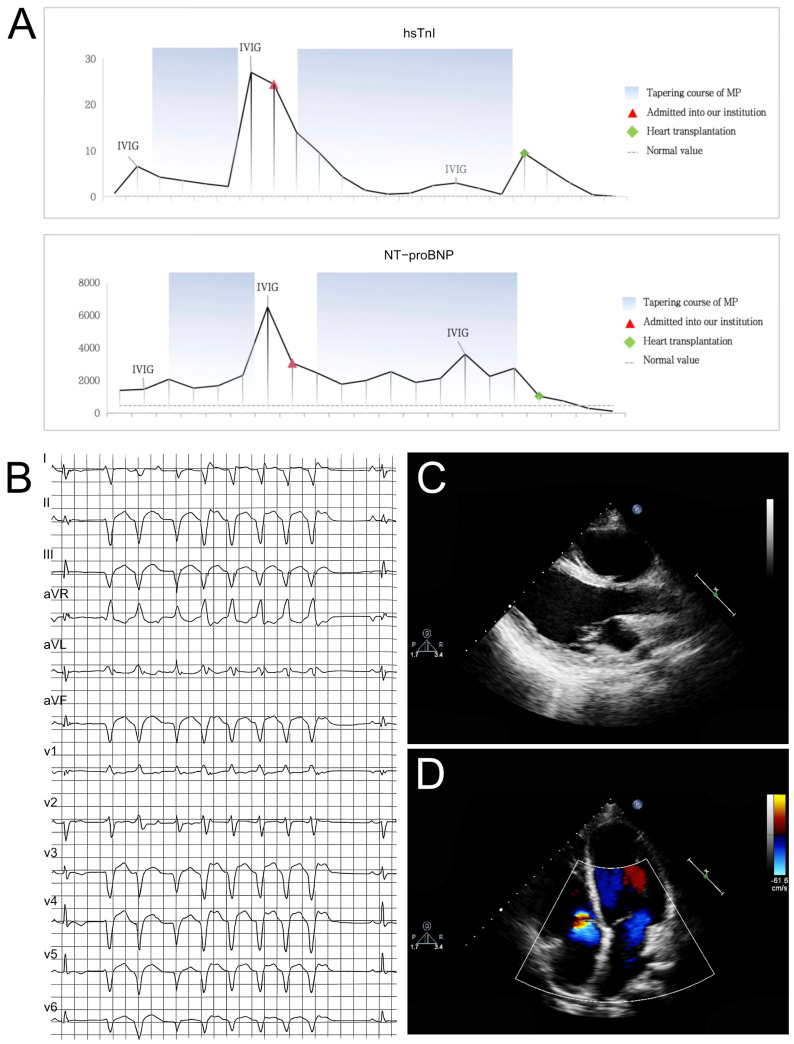
(**A**) Laboratory investigations were notable for a significant elevation in both hs-TnI and NT-proBNP levels. (**B**) ECG at presentation showed multifocal and polymorphic premature ventricular complexes, brief runs of non-sustained ventricular tachycardia. (**C**,**D**) Echocardiography revealed cardiac enlargement (which progressed from being predominantly left-sided to global involvement), thinning of ventricular wall, impaired ventricular systolic function (progressing from isolated left ventricular dysfunction to biventricular involvement). Additional findings included severe atrioventricular valvular regurgitation and pericardial effusion.

Laboratory investigations revealed electrolyte disturbance with hyponatremia, elevated cardiac troponin I (cTnI), and markedly elevated N-terminal pro B-type natriuretic peptide (NT-proBNP) (Figure 1A). Notably, comprehensive hematological, hepatic, renal, and thyroid function tests were within reference ranges. Additional parameters, including pyruvate, lactate, and ammonia concentrations, alongside urinalysis and fecal examination, showed no significant abnormalities. Autoimmune serology was notable for weakly positive anti-SS-A/R52 antibodies. Thoracic, cervical, and cerebral computed tomography revealed bilateral pulmonary inflammation with interstitial changes, marked cardiomegaly and remarkable bilateral ventricular dilatation.

Electrocardiography revealed abnormalities including ST-T changes, low voltage and ventricular arrhythmias (Figure 1B). Echocardiography demonstrated left ventricular dysfunction, left ventricular enlargement with altered echogenicity, with the left ventricular end-diastolic diameter (LVEDD) as 49 mm, severe mitral regurgitation and ventricular systolic dysfunction recording a decreased left ventricular ejection fraction (LVEF) as 48% (Figure 1C,D). Moreover, confirmed these findings and showed late gadolinium enhancement (LGE) involving the epicardial and mid-myocardial layers. Coronary computed tomography angiography revealed just a superficial myocardial bridge in the mid-left anterior descending artery.

### 3.3. Initial Medical Therapy

The preliminary diagnosis of fulminant myocarditis had been established. The patient received standard immunosuppressive therapy consisting of intravenous immunoglobulin (IVIG, 2 g/kg) and high-dose methylprednisolone pulse therapy followed by a tapering regimen. Concurrent treatment included optimized guideline-directed heart failure therapy, amiodarone for arrhythmia management and standard supportive care measures. Initially, aggressive management resulted in clinical improvement with a significant reduction in troponin-I, cardiac enzymes, and NT-proBNP levels. However, during the steroid tapering phase, a rebound elevation in cTnI, myocardial enzymes, and NT-proBNP was observed. A second course of IVIG therapy failed to demonstrate clinical efficacy. Serial echocardiographic assessments revealed progressive deterioration in left ventricular systolic function, with LVEF declining from 48% to 30% over a two-week period.

**Figure 2:**
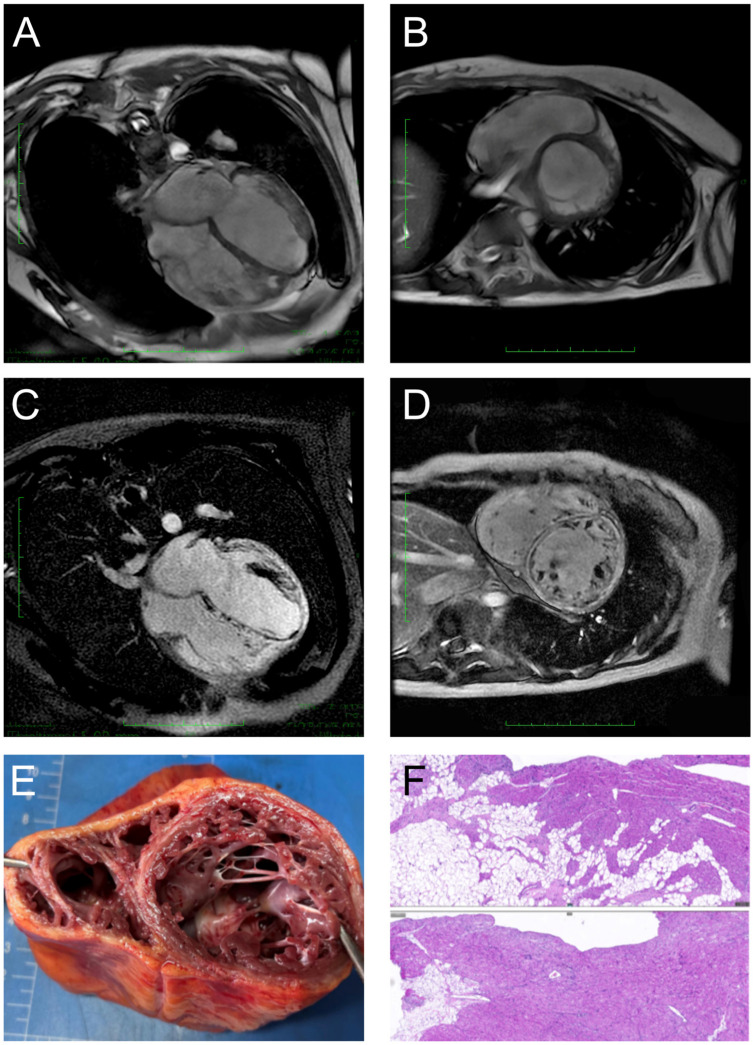
(**A**,**B**) Cardiac MRI showed biventricular dilation and segmental wall motion abnormalities. The scale bar indicated 1 cm per minor stick. (**C**,**D**) LGE images revealed a characteristic circumferential pattern of left ventricular enhancement. The scale bar indicated 1 cm per minor stick. (**E**,**F**) Histopathological examination of the explanted heart revealed the following key findings: Biventricular myocardium exhibited multifocal patchy inflammatory infiltrates, composed predominantly of lymphocytes and histiocytes, accompanied by interstitial fibrosis. The right ventricular myocardium was significantly diminished and largely replaced by adipose tissue.

The addition of oral mycophenolate mofetil provided only transient clinical benefit. Despite intensive therapy, the patient’s condition deteriorated rapidly. Follow-up echocardiography demonstrated progressive left ventricular dilatation with persistently depressed systolic function (LVEF declining from 30% to 20%). Notable morphological changes included progressive LV wall thinning, heterogeneous myocardial echotexture, and increased endocardial echogenicity. Cardiac MRI revealed dramatic disease progression with biventricular dilatation (LVEDD = 61.3 mm), severe LV systolic dysfunction (LVEF = 14%, Figure 2A,B), and extensive circumferential LV LGE (Figure 2C,D). Concurrently, the patient developed early manifestations of cardiogenic shock, requiring vasoactive pharmacological support to maintain hemodynamic stability.

### 3.4. Molecular Results

**Figure 3:**
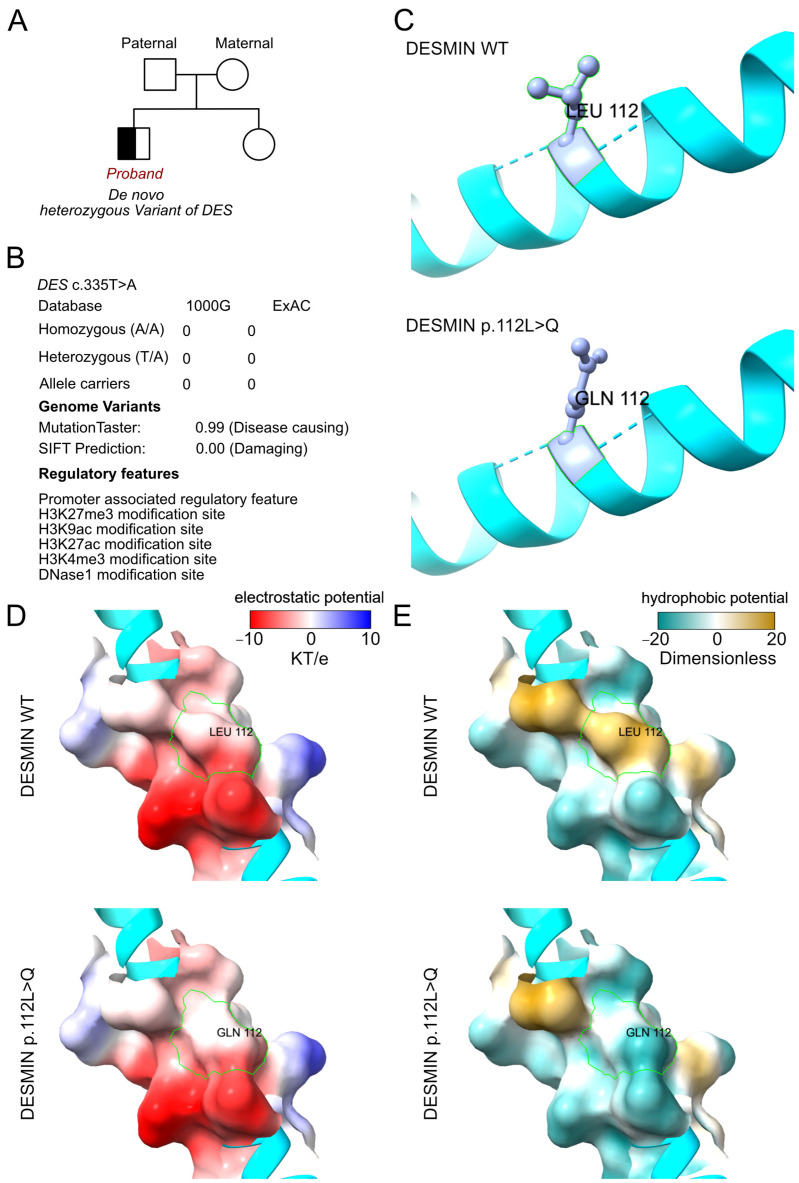
**The molecular features of DESMIN.** (**A**) The proband exhibited a de novo heterozygous variant of *DES* (c.335T>A, p.L112Q). (**B**) The variant of *DES* c.335T>A had never been reported in ExAC and 1000G databases, which was predicted to be protein-damaging by MutationTaster and SFIT. (**C**) The protein structure of DESMIN had been built by AlphaFold3 based on the wildtype and mutant sequencing. Then, the residue relationship around the mutant site indicates the structure presented due to the variant of DESMIN p.L112Q. (**D**,**E**) The electrostatic potential and hydrophobic potential changes based on the DESMIN variant of p.L112Q.

WES had been performed for this proband and validated by Sanger sequencing among her parents and sister. According to the analyzed results from WES, a de novo heterozygous variant had been identified as c.335T>A (p.L112Q) of the *DES* gene (Figure 3A). And the variant of *DES* c.335T>A had never been reported in ExAC and 1000G database (Figure 3B). In detail, we identified all the potential cardiomyopathy-associated variants and validated pathogenic or likely pathogenic variants. And no additional cardiomyopathy-associated variant had been identified. In addition, we had excluded other potential variants involved in cardiovascular, metabolic and muscular disorders. Then the SWSIS model tool was used to establish the protein crystal structure and analyze the mutant site of DESMIN (Figure 3C) [13,14]. The amino acid changed at the 112 site according to the variant of DESMIN p.L112Q. However, the variant did not alter the surrounding residues and hydrogen bond (Figure 3C). Moreover, according to the analysis results of ChimeraX software v 1.11.1, after L112Q substitution, the hydrogen bond network of DESMIN protein (green dashed line) did not change, but the contact points with water molecules (yellow dashed line) significantly decreased. The L112Q substitution introduces a decrease in the hydrophobicity of this site and may reduce its interaction with the surrounding hydrophobic groups, thereby affecting the overall conformation and stability of the protein (Figure 3D,E).

### 3.5. Final Diagnosis and Treatment

Given the irreversible nature of the cardiomyopathy and progressive hemodynamic compromise, the patient underwent successful orthotopic heart transplantation. The patient underwent heart transplantation one month after being diagnosed with ACM, which revealed an unsatisfactory prognosis of an aggressive *DES* variant-induced ACM with severe heart failure. The immediate postoperative course was favorable, with extubation achieved within 5 h post-transplantation. The early recovery period was complicated by cytomegalovirus infection at one month post-transplantation, which was effectively managed through targeted antiviral therapy and optimization of the immunosuppressive regimen. At the 12-month follow-up, the patient demonstrated excellent clinical recovery.

Histopathological examination of the explanted heart provided definitive diagnostic confirmation. The pathological findings established a diagnosis of biventricular ACM, characterized by extensive fibrofatty replacement of the ventricular myocardium with predominant right ventricular involvement and significant fibrosis of the left ventricular endocardium and interventricular septum (Figure 2E,F). Notably, histological analysis also revealed multifocal lymphohistiocytic inflammatory infiltrates throughout the myocardium.

## 4. Discussion

This case underscores the critical importance of early recognition of ACM presenting with “hot phases” and highlights the potentially life-threatening consequences of diagnostic delay. Given the substantial clinical complexity and symptomatic overlap between ACM with hot-phase presentations and myocarditis, no single gold-standard diagnostic modality exists. A comprehensive multiparametric diagnostic approach integrating genetic, electrocardiographic, morphofunctional, and histopathological data is essential for accurate differentiation [1,3,10,15,16,17]. Family history evaluation remains paramount given the genetic predisposition underlying ACM [5,18].

Cardiac MRI represents a fundamental non-invasive imaging modality for evaluating both ACM and myocarditis, effectively demonstrating myocardial inflammation, necrosis, and edema while characterizing biventricular volumetric and functional parameters [7,12]. Comparative analysis reveals that ACM patients typically demonstrate reduced myocardial edema, more extensive LGE, increased left ventricular volumes, and a higher prevalence of wall thinning and regional wall motion abnormalities compared to myocarditis patients. Additionally, subepicardial LGE distribution patterns may suggest ACM, whereas intramural patchy LGE patterns are highly suggestive of myocarditis. In our case, ACM was suspected only after follow-up CMR demonstrated extensive circumferential left ventricular LGE coinciding with rapidly progressive systolic dysfunction and wall thinning.

Pathogenic variants in genes encoding desmosomal proteins, phospholamban, and filamin-C constitute established genetic substrates for ACM. Consequently, molecular genetic criteria were incorporated into diagnostic guidelines in 2010 as major diagnostic criteria. Over the past decade, multigene panel testing has emerged as an essential diagnostic component in suspected ACM cases. Among ACM patients presenting with hot-phase episodes, *DSP* mutations predominate (69%), followed by *PKP2* and *DSG2* variants (17%) [19,20,21,22]. The *DES* gene encodes DESMIN, a type III intermediate filament protein predominantly expressed in muscle cells that maintains mechanical integrity and cellular stability [23,24,25]. The association between *DES* gene mutations and ACM was first proposed by Van Tintelen et al. in 2009 [26]. *DES* mutations account for approximately 1% of ACM cases and are associated with predisposition for left heart involvement and diverse clinical phenotypes. Limited data suggest an elevated incidence of major adverse cardiac events in patients harboring pathogenic or likely pathogenic *DES* mutations. However, pleiotropic presentations and small cohort sizes have constrained comprehensive clinical phenotype and outcome characterization.

In our case, a *DES* variant was identified, ultimately supporting the diagnosis of ACM. The proposed mechanism by which *DES* variants contribute to ACM involves compromise of cytoskeletal–desmosomal network integrity, rendering cardiomyocytes more susceptible to mechanical stress-induced damage and apoptosis, thereby initiating a pathogenic cascade of programmed cell death, fibrofatty repair, and inflammatory activation.

In patients with early-stage ACM presenting during the “hot phase,” the primary objective of early diagnosis is to capitalize on the reversible window of immune activation, interrupt the multi-mechanistic injury cascade, and prevent irreversible myocardial damage, thereby potentially improving clinical outcomes [4,27]. Recent evidence suggests that such patients may benefit from timely and adequate immunosuppressive therapy, which could attenuate disease progression and defer the need for heart transplantation [1,6]. In the present case, standardized therapy including immunosuppression was initiated promptly following multidisciplinary consultation. Although immunotherapy successfully suppressed inflammation and elicited short-term clinical responses, transient inflammatory control failed to halt the progression of myocardial injury. Despite the absence of treatment delay, myocardial damage continued to advance, accompanied by rapid and persistent deterioration of cardiac function. Early recognition of ACM would have facilitated more accurate prognostication, obviated repeated courses of potentially unnecessary aggressive immunotherapy and premature guideline-directed medical therapy (GDMT), and potentially enabled earlier consideration of left ventricular assist device (LVAD) implantation [28,29].

Immunotherapy demonstrated short-term efficacy in this case, as evidenced by decreased BNP and troponin levels, as well as alleviation of chest pain. However, heart transplantation remained unavoidable due to the rapid progression of myocardial injury. We propose the following potential underlying mechanisms to account for the limited therapeutic response. (1) Disease heterogeneity and undefined immune phenotyping. ACM exhibits considerable heterogeneity and may be stratified into distinct subtypes based on underlying pathogenic mechanisms, with substantial interindividual variation in immune activation patterns. Currently, targeted therapeutic strategies are lacking, and immunotherapies are applied empirically. The absence of precision therapy tailored to individual immune phenotypes represents a potential contributor to suboptimal treatment efficacy [1,4,27]. (2) Convergence of immune and non-immune pathological mechanisms. Beyond immune-mediated inflammation, the “hot phase” of ACM is characterized by multiple concurrent non-immune pathological processes, including cardiomyocyte apoptosis, oxidative stress, microcirculatory dysfunction, and arrhythmia-induced secondary myocardial injury. Immunotherapy selectively suppresses immune-mediated inflammation but fails to address these parallel injury mechanisms [9]. (3) Persistence of underlying genetic defects. Inflammation in ACM represents a secondary phenomenon triggered by intrinsic genetic defects in cardiomyocytes [12,30]. Current immunotherapies are unable to correct these fundamental genetic abnormalities, resulting in recurrent immune activation and sustained disease progression. Thus, for “hot-phase” ACM, immunotherapy could benefit short-term clinical outcomes, providing a time window for precise diagnosis and therapeutic strategy application, including GDMT and LVAD. However, due to the basis of such a disease, recurrent administration of immunotherapy should be avoided.

The prominent pathological features of this case are myocardial replacement by adipose tissue in both ventricles with interstitial fibrosis, with some inflammation infiltration, which was identified by the finding of lymphocyte infiltration. Therefore, the pathological findings support the diagnosis of ACM, and the inflammation infiltration could explain the presentation of “hot phase”. As for inflammatory cardiomyopathy, the main pathological manifestation is the presence of significant inflammatory cell infiltration (predominantly lymphocytes) within the myocardium, accompanied by degeneration, necrosis of cardiomyocytes, and interstitial fibrosis. Consequently, distinguishing between inflammatory cardiomyopathy and inherited cardiomyopathies, especially for “hot-phase” ACM, should be carefully evaluated.

## 5. Conclusions

Our management of this case elucidates three critical diagnostic and therapeutic insights. First, it exemplifies the substantial diagnostic challenge posed by recurrent myocarditis-like “hot-phase” presentations in ACM, which frequently result in initial misdiagnosis and delayed definitive care. Second, it emphasizes the necessity for heightened clinical awareness regarding early imaging characteristics of ACM, particularly subtle morphofunctional abnormalities that may precede overt structural changes. Third, our findings suggest that *DES* variants may constitute a novel genetic substrate for a distinct subset of ACM cases characterized by hot-phase presentations, thereby expanding the recognized genetic spectrum of this condition. Early recognition of these clinical patterns is essential for timely diagnosis, appropriate risk stratification, and implementation of targeted therapeutic interventions to optimize patient outcomes and facilitate cascade family screening.

## Data Availability

Data sets used in this study are available from the corresponding author upon reasonable request.
